# A Comparison of the Anti-Tumor Effects of a Chimeric *versus* Murine Anti-CD19 Immunotoxins on Human B Cell Lymphoma and Pre-B Acute Lymphoblastic Leukemia Cell Lines

**DOI:** 10.3390/toxins3040409

**Published:** 2011-04-06

**Authors:** Lydia K. Tsai, Laurentiu M. Pop, Xiaoyun Liu, Ellen S. Vitetta

**Affiliations:** The Cancer Immunobiology Center, University of Texas Southwestern Medical Center at Dallas, 6000 Harry Hines Blvd, Dallas, TX 75390, USA; Email: lydiaktsai@gmail.com (L.K.T.); laurentiu.pop@utsouthwestern.edu (L.M.P.); xiaoyun.liu@utsouthwestern.edu (X.L.)

**Keywords:** chimerization, anti-CD19, ricin A chain

## Abstract

Precursor B cell acute lymphoblastic leukemia (pre-B ALL) affects five to six thousand adults and almost three thousand children every year. Approximately 25% of the children and 60% of the adults die from their disease, highlighting the need for new therapies that complement rather than overlap chemotherapy and bone marrow transplantation. Immunotherapy is a class of therapies where toxicities and mechanisms of action do not overlap with those of chemotherapy. Because CD19 is a B cell- restricted membrane antigen that is expressed on the majority of pre-B tumor cells, a CD19-based immunotherapy is being developed for ALL. In this study, the anti-tumor activities of immunotoxins (ITs) constructed by conjugating a murine monoclonal antibody (MAb), HD37, or its chimeric (c) construct to recombinant ricin toxin A chain (rRTA) were compared both *in vitro* using human pre-B ALL and Burkitt’s lymphoma cell lines and *in vivo* using a disseminated human pre-B ALL tumor cell xenograft model. The murine and chimeric HD37 IT constructs were equally cytotoxic to pre-B ALL and Burkitt’s lymphoma cells *in vitro* and their use *in vivo* resulted in equivalent increases in survival of SCID mice with human pre-B ALL tumors when compared with control mice.

## 1. Introduction

Precursor B cell acute lymphoblastic leukemia (pre-B ALL) affects five to six thousand adults and almost three thousand children every year. Despite the development of more sensitive detection methods and more effective treatment options, approximately 25% of children [1,2] and 60% of adults [1,3] with pre-B ALL are not cured by conventional therapy. First line chemotherapy consists of cyclophosphamide, vincristine, adriamycin and dexamethasone (CVAD) [4]. CVAD is associated with acute and chronic toxicities including myelosuppression, cardiac toxicity, nausea, vomiting, infections, *etc.* [5]. If the disease relapses, doses of chemotherapy are increased, sometimes resulting in short term responses and always resulting in increased toxicity. Patients who relapse 2^nd^ line therapy often undergo allergenic or autologous bone marrow transplantation, which is associated with additional toxicity and sometimes, but rarely, durable responses. Effective MAb therapy for pre-B ALL would be a significant advantage over chemotherapy or radiotherapy because MAbs would selectively kill the leukemic cells, resulting in less toxicity. In addition, toxicity from a MAb- based therapy is unlikely to overlap toxicity from traditional chemotherapy and could thus be combined with chemotherapy or used as consolidation therapy immediately following chemotherapy without risk of exacerbating chemotherapy induced toxicity.

Several MAb- based therapies have been approved by the Food and Drug Administration (FDA) for lymphoma or leukemia. Three MAb-based therapies, all of which target CD20, have been approved for the treatment of non-Hodgkin’s lymphoma. One of these is unconjugated, and two are conjugated to radionuclides [6]. CD20 is a B cell specific transmembrane protein; it is expressed only on mature B lymphocytes and not on pro-B or pre-B cells [7,8] and therefore would not be effective in pre-B ALL. Campath, an anti-CD52 MAb is approved for use in B cell chronic lymphocytic leukemia but would also not be effective in pre-B cell ALL. Furthermore, there are no MAb-based therapies that have been FDA-approved for the treatment of childhood malignancies. These therapies confirm the potential of MAb based therapies for treatment of leukemia and lymphoma.

CD19 is also a transmembrane glycoprotein that is restricted to B cell expression in both mouse and humans [9]. It is expressed on B cells following the differentiation of pluripotent stem cell into committed B lymphocytes and is expressed until terminal differentiation of lymphocytes into plasma cells [10]. Thus, CD19 is expressed on pre-B cells (including pre-B ALL cells) while CD20 is not, and as compared to CD20, CD19 expression persists longer on B cells during their maturation. More lymphoid malignancies express CD19 than CD20 [10,11]. In addition, unlike CD20, CD19 is rapidly internalized [10,12] which makes it a more attractive target for an IT. 

One of the most commonly used toxins for chemical construction of ITs is RTA. Its native form is *N*-glycosylated and hence mannose and fructose receptors on liver cells can recognize and bind the RTA protein of iTs, thus shortening their half lives and causing liver damage. For this reason, RTA has been chemically deglycosylated (dgRTA) before its conjugation [13]. To avoid the problem of working with ricin, a recombinant RTA devoid of all carbohydrate moieties is now expressed in bacteria [14]. Despite some difference in their structure, the function and the biological activities of dgRTA and rRTA are the same [15].

*In vitro*, the anti-CD19 IT, HD37-dgRTA, is effective in killing Burkitt’s lymphoma cell lines, pre-B ALL cell lines and leukemic cells from children with pre-B ALL [16,17]. *In vivo*, HD37-dgRTA prolonged the survival of SCID mice that had been injected with human non-Hodgkin’s lymphoma (NHL) cells or pre-B ALL cells compared with the survival of control mice injected with saline or an isotype-matched control IT [18,19,20,21]. Treatment with a mixture of HD37-dgRTA and RFB4-dgRTA, an anti CD22 IT, was more effective than either IT alone [21]. 

The anti-tumor efficacy of HD37-dgRTA in adults with NHL was demonstrated in Phase I clinical trials. In addition, HD37-dgRTA was used in combination with RFB4-dgRTA in children with pre-B ALL [22,23,24]. Three of 17 children experienced complete remissions and the combination was well tolerated. Although these studies held promising results for the anti-tumor efficacy of the ITs, approximately 25–30% of patients made human anti-mouse antibodies (HAMA) against HD37, which prevented additional courses of treatment in those patients. Since many of these patients had experienced significant decreases in tumor burden, the inability to administer repeat courses of treatment was clinically very significant. 

In this study our goals were to construct a chimeric HD37 MAb (cHD37), containing the variable regions of the murine HD37 and the human constant IgG1κ region, and then to construct an IT by conjugating cHD37 to rRTA (cHD37-rRTA). This IT should be less immunogenic in humans although there might still be a response to RTA. We compared the *in vitro* activity of cHD37-rRTA with HD37-rRTA using human Burkitt’s lymphoma and pre-B ALL cell lines. They were equally cytotoxic *in vitro*. We then compared the *in vivo *activity of cHD37-rRTA with HD37-rRTA in SCID mice xenografted with a human pre-B ALL cell line (SCID/NALM-6 mice). The anti-tumor activity of cHD37-rRTA and HD37-rRTA were equally effective in prolonging the survival of SCID/NALM-6 mice as compared to PBS and an isotype-matched control IT. 

## 2. Materials and Methods

### 2.1. Construction and Expression of the cHD37 MAb

The heavy and light chains of cHD37 were generated using vectors containing the human IgG1 and kappa constant region domains, pAH4604 and pAG4622. These were a generous gift from Dr. Sherrie Morrison [25]. The heavy and light variable domains of HD37 were separately amplified by PCR using cDNA prepared from HD37 hybridoma cells with primers that annealed in their respective inferred leader peptides and their constant domains. After verifying the accuracy of the variable domain genes, primer pairs A/B and C/D were used to amplify HD37 genes for insertion into antibody expression vectors. The primers are as follows: A, 5’-GGGTCTAGATATCCACCATGGGATGGAGCTTGATCTTTCTCTT-3’; B, 5’-GTCTAGGAATTCGCTAGCTGAGGAGACGGTGACTGAGG-3’; C, 5’-GGGTCTAGATATCCACCATGGAGACAGACACACTCCTGCTATGGG-3’; and D, 5’-GTCTAGGAATTCGTCGACTTACGTTTGATTTCCAGCTTGGTGC-3’.

The heavy and light chain constructs were co-transfected into SP2/0-AG14 cells (ATCC, Manassas, VA) using Lipofectamine 2000 (Invitrogen, Carlsbad, CA). Stable transfectants were selected using Dulbecco’s Minimal Essential medium (DMEM) medium (Sigma, St. Louis, MO) supplemented with 10% heat inactivated fetal bovine serum (FBS) (Hyclone, Logan, UT), 2 mM glutamate, and 10 mM L-histidinol (Sigma, St. Louis, MO). Positive clones were screened using human Ig-specific enzyme linked immunosorbent assays (ELISAs).

### 2.2. Purification of cHD37

Proteins in the cell culture supernatant were precipitated at 4 °C with ammonium sulfate at 50% saturation, dissolved in distilled water and dialyzed overnight against PBS, pH 7.5. The dialysate was affinity-purified on Protein G-Sepharose (Amersham Biosciences, Piscataway, NJ), and the bound proteins were eluted with 0.1 M Glycine-HCl-NaN_3_ buffer, neutralized and dialyzed overnight against PBS, pH 7.5. The dialysate was concentrated, filter-sterilized, and stored at 4 °C.

### 2.3. Preparation of ITs

HD37 and cHD37 were chemically conjugated to either dgRTA [16,26] or rRTA [15] using the linker 4-succinimidyl-oxycarbonyl-α-methyl-α-(2-pyridyldithio)-toluene (SMPT) (Pierce, Rockford, IL) and purified as previously described [27,28]. Briefly, SMPT dissolved in dimethylformamide (Sigma) was added to a solution of antibody (5 mg/mL) to give a final molar ratio of linker to antibody of 5:1. After incubation for 1 h at room temperature, the solution was passed through a column of Sephadex G-25 (Amersham Biosciences) in PBS. The derivatized protein contained 1.5–2.5 α-methyl-α(2-pyridyldithio)toluoyl groups as determined by spectrophotometry. The derivatized protein was then mixed with freshly reduced rRTA using 0.5 mg rRTA per mg of MAb, and maintained under sterile conditions for 36 h at room temperature while the reaction proceeded. The resulting IT was purified by affinity chromatography, first by binding the IT and unconjugated rRTA onto Blue Sepharose (Amersham Biosciences) in 0.05 M phosphate buffer, at pH 7.0. Then, free rRTA and IT were eluted with 1 M NaCl in 0.05 M phosphate buffer, pH 7.0. The eluate was chromatographed on Superdex 200 (Amersham Biosciences) in PBS. The first peak, which contained the IT, was collected, concentrated by ultrafiltration, sterilized by filtration and stored in aliquots at 4 °C. HD37-dgRTA and RFT5-dgRTA (isotype-match control IT) were constructed in the same manner using dgRTA instead of rRTA as previously described [16,26].

### 2.4. Sodium Dodecyl Sulfate Polyacrylamide Gel Electrophoresis (SDS-PAGE)

The purity and molecular weights of the ITs were analyzed by 4–15% SDS-PAGE (Amersham Biosciences) using a PhastSystem™ (Amersham Biosciences). The gel was stained with PhastGel Blue R (Amersham Biosciences).

### 2.5. Cells

Cell lines include the CD19-expressing human Burkitt’s lymphoma cell lines: Daudi, Namalwa, and Raji; the human pre-B ALL cell line: NALM-6; and the human FcγRI-expressing cell line: U937. Cells were obtained from ATCC (Manassas, VA) and maintained in culture by serial passages in complete medium consisting of RPMI-1640 (Sigma) supplemented with 10% heat-inactivated FBS and 2 mM L-glutamine.

### 2.6. Antigen-Binding Activity of ITs and MAbs

One million cells were incubated with dilutions of ITs or MAbs (0.01–10 μg/mL) followed by FITC-labeled goat anti-human Ig (GAHIg) under saturating conditions and analyzed on a FACSCalibur (Becton Dickinson, San Jose, CA). The percentage of positive cells was plotted against the concentration of IT or MAb.

### 2.7. [^3^H]Thymidine Incorporation

Tumor cells cultured in complete medium (5 × 10^4^ cells/100 μL) were plated in 96-well plates and incubated for 48 h at 37 °C with 100 μL of different concentrations of the ITs (ranging from 10^−8^ to 10^−13^ M) or controls. The cells were then pulsed for 4 h at 37 °C with 0.5 μCi [^3^H]thymidine (Amersham Biosciences), harvested and counted in a liquid scintillation spectrometer. The percentage reduction in [^3^H]thymidine incorporation at different concentrations of ITs compared with thymidine incorporation in the cells to which controls were added was used to quantitate the cytotoxic effect (expressed as the IC_50_).

### 2.8. Therapy of SCID/NALM-6 Mice

Six to nine weeks old female SCID-SCRF-M mice (Taconic, Germantown, NY) were inoculated intravenously in the tail veins with 5 × 10^6^ NALM-6 cells in 0.1 mL sterile PBS, pH 7.4. After 24 h, groups of 5 mice were injected intraperitoneally with PBS or different ITs in four equal injections on days 1 to 4 after tumor inoculation. The treatment groups were as follows: (1) PBS, pH 7.4; (2) 4.7 μg/g HD37-rRTA; (3) 4.7 μg/g cHD37-rRTA; (4) 4.7 μg/g HD37-dgRTA; and (5) 4.7 μg/g RFT5-dgRTA. The dose for the IT treatment was 40% of the LD_50_ of HD37-dgRTA based on previous studies [17,20]. Mice were followed daily and were sacrificed at the onset of paralysis, a clinical symptom which accurately predicts death. The mean paralysis time (MPT) was taken as the end point. MPT curves were compared using the Wilcoxon matched-pairs log-rank test and were considered to be significantly different when *p* < 0.05.

## 3. Results

### 3.1. Construction, Expression and Purification of cHD37

**Figure 1:**
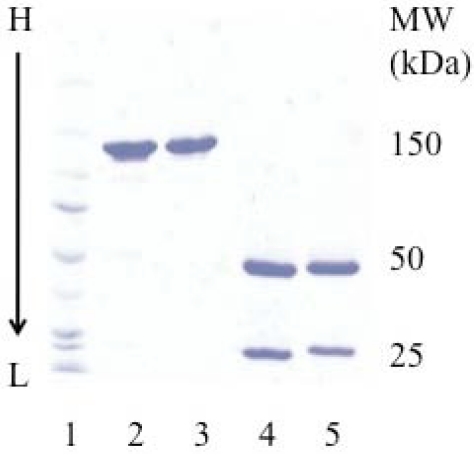
SDS-PAGE analysis of the purified cHD37 and HD37 MAbs. A 4–15% gradient gel was performed under non-reducing (lanes 2 and 3) and reducing (lanes 4 and 5) conditions. Lane 1: molecular weight markers; lanes 2 and 4: murine HD37; lanes 3 and 5: cHD37. This is one representative gel from three experiments.

cHD37 was expressed and purified to homogeneity. The purity and molecular weight were assessed by SDS-PAGE. There were no differences in the purity or the molecular weight of the recombinant construct and the murine HD37 used as a control (Figure 1).

### 3.2. Preparation of ITs

**Figure 2:**
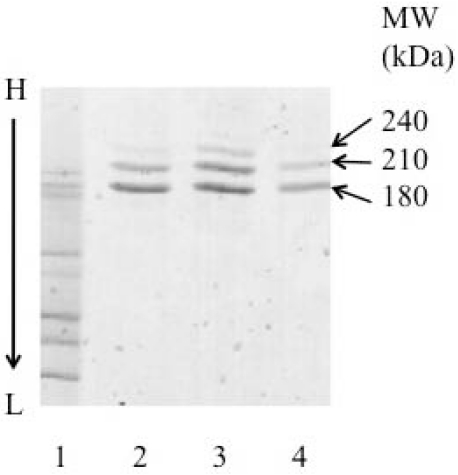
SDS-PAGE analysis of the purified cHD37-rRTA and HD37-rRTA ITs.A 4–15% gradient gel was performed under non-reducing conditions. Lane 1, molecular weight markers; lane 2: cHD37-rRTA; lane 3: HD37-rRTA; lane 4: HD37-dgRTA. This is one representative gel from three experiments.

ITs were constructed by coupling the recombinant or murine HD37 MAbs to rRTA or dgRTA using the heterobifunctional linker, SMPT [27,28]. This linker generates one or more hindered disulfide bonds between the MAb and RTA. SDS-PAGE densitometric analysis of the ITs showed that >60% of the protein was found in the major band at 180 kDa (one molecule of IgG and one molecule of rRTA). In addition there were two minor bands (~30% and 10%) corresponding to ITs with two or three molecules of rRTA or dgRTA per molecule of IgG (210 and 240 kDa, respectively) for the ITs (Figure 2).

### 3.3. Binding of ITs and MAbs to CD19^+^ Cells

**Figure 3:**
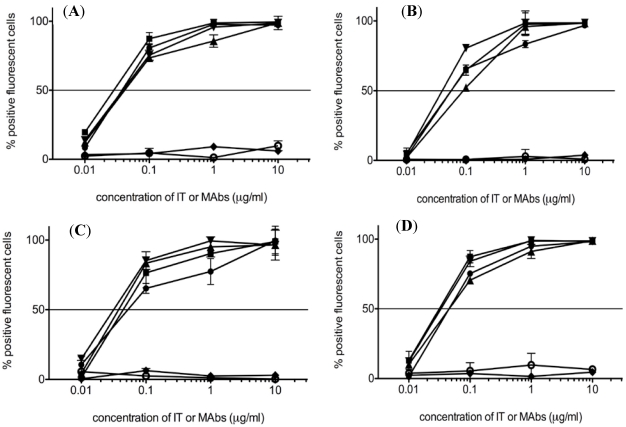
Binding of HD37 IT and MAb constructs to CD19^+^ cell lines. The ability of the ITs and MAbs to bind CD19^+^ (**A**) NALM-6, (**B**) Daudi, (**C**) Namalwa, and (**D**) Raji cells was assessed by flow cytometry. Diamonds: RFT5-dgRTA; open circles: MOPC-21(control); squares: cHD37; circles: HD37; triangles: HD37-rRTA; inverted triangles: cHD37-rRTA. Data represent means ± SD of three experiments.

To determine whether chimerization of HD37 affected its binding to target cells, HD37, HD37-rRTA, cHD37, and cHD37-rRTA were evaluated for binding to CD19^+^ cells by flow cytometry (Figure 3). All four compounds bound equally well in a dose dependent manner to NALM-6, Daudi, Namalwa and Raji cells. Neither the isotype-matched control myeloma protein, MOPC-21 nor the control IT, RFT5-dgRTA, bound to the cells. These results indicate that chimerization of HD37 did not affect the binding of the MAb to CD19.

### 3.4. Cytotoxicity of the ITs *in Vitro*

**Figure 4:**
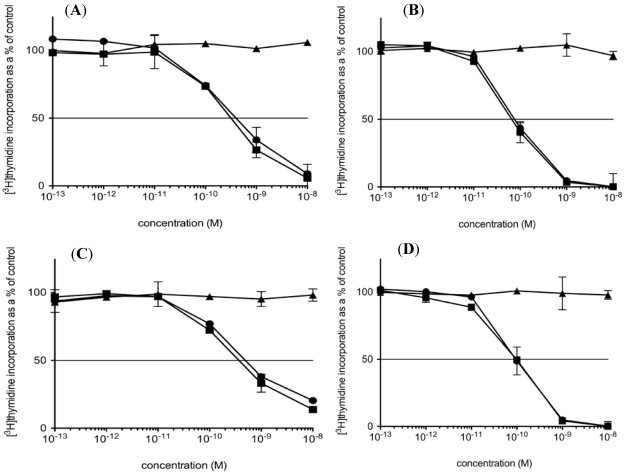
Cytotoxicity of HD37 IT constructs on CD19^+^ cells lines.The cytotoxicity of the HD37 IT constructs were evaluated by [^3^H]thymidine incorporation on (A) NALM-6, (B) Daudi, (C) Namalwa, and (D) Raji cells. Triangles: RFT5-dgRTA; squares: cHD37-rRTA; circles: HD37-rRTA.

Cytotoxicity of HD37-rRTA, cHD37-rRTA and the control IT, RFT5-dgRTA, to human pre-B ALL NALM-6 cells is shown in Figure 4A. Both HD37 IT constructs killed the cells with equal efficiency (IC_50_ of HD37-rRTA = 4 × 10^−10^ M; IC_50_ of cHD37-rRTA = 3 × 10^−10^ M), while the control IT had no effect. Cytotoxicity was also assessed in human Burkitt’s lymphoma cell lines, and both HD37 ITs killed Daudi (IC_50_ of HD37-rRTA = 7 × 10^−11^ M; IC_50_ of cHD37-rRTA = 6 × 10^−11^ M), Namalwa (IC_50_ of HD37-rRTA = 5 × 10^−10^ M; IC_50_ of cHD37-rRTA = 3.5 × 10^−10^ M), and Raji (IC_50_ of HD37-rRTA = 10^−10^ M; IC_50_ of cHD37-rRTA = 10^−10^ M) cell lines equally well (Figure 4B–D). These results indicate that the chimerization of the MAb did not affect the cytotoxicity of the IT that it was prepared with.

### 3.5. Therapeutic Efficacy of the ITs in SCID/NALM-6 Mice

**Figure 5:**
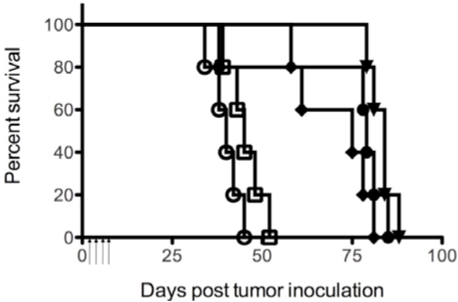
Therapeutic efficacy of the HD37 IT constructs in SCID mice with NALM-6 tumor cell xenografts. SCID/NALM-6 mice were treated on days 1 through 4 after tumor inoculation with the following ITs (5 mice per treatment group): open circles: PBS; open squares: RFT5-dgRTA; inverted triangles: HD37-rRTA; diamonds: cHD37-rRTA; circles: HD37-dgRTA. Arrows indicate treatment days (1–4). The graphs are representative of three separate experiments.

Since HD37-rRTA and cHD37-rRTA exhibited cytotoxic activity against NALM-6 cells *in vitro*, and previous studies using SCID/NALM-6 mice demonstrated anti-tumor activity of HD37-dgRTA [18], SCID mice xenografted with NALM-6 cells were chosen to compare the *in vivo* anti-tumor activity of HD37-dgRTA, HD37-rRTA and cHD37-rRTA. SCID mice were injected with 5 × 10^6^ NALM-6 tumor cells on day 0. Groups of 5 mice were then treated with 4.7 μg/g of IT on days 1–4 post tumor inoculation. As compared to PBS (*p* < 0.006), the RFT5-dgRTA control (*p* < 0.017), all three anti-CD19 ITs significantly extended the MPT of SCID/NALM-6 mice and however, there was no difference in the MPT of the groups treated with HD37-dgRTA, HD37-rRTA, or cHD37-rRTA (*p* < 0.233) (Figure 5). These results show that chimerization of the HD37 has not affected the *in vivo* potency of the IT against NALM-6 tumors in SCID mice. 

## 4. Discussion

Previous studies have shown that an anti-CD19 IT, HD37-dgRTA, is effective in prolonging the survival of SCID mice xenografted with human pre-B ALL or NHL tumors either alone or in combination with an anti-CD22 IT and/or chemotherapy [18,19,20,21]. Phase I clinical trials in humans with lymphoma and leukemia have demonstrated the anti-tumor efficacy of HD37-dgRTA either alone or in combination with an anti-CD22 IT [22,23,24]. 

The goal of this study was to construct cHD37 MAb and cHD37-rRTA IT and to compare the anti-tumor activity of cHD37-rRTA with HD37-rRTA. Our study showed that chimerization of HD37 did not affect its binding activity, its *in vitro *cytotoxic activity, or its *in vivo *anti-tumor efficacy. Studies in clinical trials in humans will determine whether the cHD37-rRTA will be well tolerated in patients and whether HAMA will be generated.

In conclusion, our results indicate that the anti-tumor activity of a chimeric, recombinant anti-CD19 IT, cHD37-rRTA, is equally as efficacious as that of the murine IT, HD37-dgRTA and HD37-rRTA *in vitro *and in SCID/NALM-6 mice. Further studies in patients will be necessary to determine optimal treatment regimens and whether this IT is less immunogenic in humans. 
